# Isolation of a Hypomorphic *skn-1* Allele That Does Not Require a Balancer for Maintenance

**DOI:** 10.1534/g3.115.023010

**Published:** 2015-12-28

**Authors:** Lanlan Tang, William Dodd, Keith Choe

**Affiliations:** Department of Biology, University of Florida, Gainesville, Florida 32611

**Keywords:** genetic screen, mutant, resource

## Abstract

In *Caenorhabditis elegans*, the transcription factor SKN-1 has emerged as a central coordinator of stress responses and longevity, increasing the need for genetic tools to study its regulation and function. However, current loss-of-function alleles cause fully penetrant maternal effect embryonic lethality, and must be maintained with genetic balancers that require careful monitoring and labor intensive strategies to obtain large populations. In this study, we identified a strong, but viable *skn-1* hypomorphic allele *skn-1*(*zj15*) from a genetic screen for suppressors of *wdr-23*, a direct regulator of the transcription factor. *skn-1*(*zj15*) is a point mutation in an intron that causes mis-splicing of a fraction of mRNA, and strongly reduces wildtype mRNA levels of the two long *skn-1a/c* variants. The *skn-1*(*zj15*) allele reduces detoxification gene expression and stress resistance to levels comparable to *skn-1* RNAi, but, unlike RNAi, it is not restricted from some tissues. We also show that *skn-1*(*zj15*) is epistatic to canonical upstream regulators, demonstrating its utility for genetic analysis of *skn-1* function and regulation in cases where large numbers of worms are needed, a balancer is problematic, diet is varied, or RNAi cannot be used.

SKN-1/Nrf proteins are members of the CNC (cap ’n’ collar) family of transcription factors that are master regulators of oxidative stress resistance and longevity (Blackwell *et al.* 2015; An and Blackwell 2003; Kahn *et al.* 2008; Sykiotis and Bohmann 2008). Recent studies revealed Nrf2 as a valuable therapeutic target for cancer and neurodegenerative diseases (Joshi and Johnson 2012; Cuadrado *et al.* 2009; Zhou *et al.* 2013; Leinonen *et al.* 2014). *C. elegans* has a single functional CNC homolog named SKN-1. The genetic tractability and short lifespan of *C. elegans* has made it an important model for understanding CNC regulation and function.

Our previous studies demonstrated that SKN-1 is under direct regulation by a WD40 repeat protein named WDR-23; WDR-23 directly binds to SKN-1 to restrain its nuclear accumulation under basal conditions, presumably by recruiting the transcription factor to a ubiquitin ligase (Choe *et al.* 2009). SKN-1 also functions downstream of target of rapamycin (TOR) and insulin/IGF-1-like signaling (IIS) (Tullet *et al.* 2008; Wang *et al.* 2010; Robida-Stubbs *et al.* 2012) pathways that influence longevity, and is required for lifespan extension by dietary restriction (Tang and Choe 2015; Bishop and Guarente 2007). Regulation of SKN-1 and the mechanisms by which it influences longevity and stress resistance are highly active areas of research (Blackwell *et al.* 2015; Ewald *et al.* 2015; Steinbaugh *et al.* 2015; Tang and Choe 2015; Dresen *et al.* 2015; Chew *et al.* 2015).

Genetic tools for studying SKN-1 function include RNAi and loss-of-function alleles that introduce premature stop codons. However, these have some important limitations. RNAi in *C. elegans* is refractory in neurons and the pharynx (Kamath *et al.* 2001; Asikainen *et al.* 2005), and relies on constant feeding of a specific bacteria strain; this is problematic for studies that manipulate diet, focus on nonfeeding stages, or for testing genetic interactions with a second RNAi clone. In addition to playing a key role in stress responses and longevity, SKN-1 is also essential to embryonic development, and loss-of-function alleles are maternal effect lethal; homozygous offspring from heterozygote mothers can develop and survive for one generation, but produce no viable offspring (Bowerman *et al.* 1992). Genetic balancers that suppress recombination are used to maintain *skn-1* alleles as heterozygotes, but this hinders isolation of large populations and the strains must be checked carefully every two to three generations because recombination can occur with the balancers leading to *skn-1* allele loss (Edgley *et al.* 2006).

In this study, we identified a viable hypomorphic allele of *skn-1*(*zj15*). *skn-1*(*zj15*) is an AT to GC mutation in an intron, near an exon boundary, that disrupts splicing and strongly reduces wildtype *skn-1* mRNA levels. Functional analysis demonstrates that *skn-1*(*zj15*) reduces *in vivo skn-1* function by a degree similar to, or greater than RNAi, but is not restricted from some tissues. Importantly, a high enough fraction of homozygous *skn-1*(*zj15*) worms escape embryonic lethality so that they can be maintained without a balancer, and cultured as a large homogeneous population.

## Materials and Methods

### C. elegans strains used

Wildtype N2 Bristol, QV160 *wdr-23(tm1817); dvIs19[Pgst-4::GFP]; zjIs5* *[ttTi5605;Pgst-4::tdTomato::unc-54* *3′UTR]*, QV224 *skn-1(zj15)* outcrossed six times; *dvIs19*, QV225 *skn-1(zj15)* outcrossed four times, CL2166 *dvIs19*, QV10 *wdr-23(tm1817); dvIs19*; *vs.Is33(Pdop-3::RFP),* QV185 *skn-1(zj15)*; *wdr-23(tm1817); dvIs19*; *vs.Is33*, QV257 *skn-1(zj15)*; *zjEx116[skn-1 gDNA; Pmyo-2::tdTomato; Pmyo-3::dsRed]; dvIs19,* QV258 *skn-1(zj15); zjEx117[skn-1 gDNA; Pmyo-2::tdTomato; Pmyo-3::dsRed];* *dvIs19,* QV259 *skn-1(zj15); zjEx118[skn-1 gDNA; Pmyo-2::tdTomato; Pmyo-3::dsRed]; dvIs19*.

### Mutagenesis, screening, whole genome sequencing, and mapping

Roughly 6000 L4 to young adult QV160 worms were mutagenized in 50 mM ethyl methanesulfonate for 4 hr and allowed to recover and then lay F1 eggs on standard NA22 agar plates. Roughly 30,000 mutagenized F1 worms were divided among 15 10-cm NA22 plates, and allowed to lay a total of about 300,000 F2 worms. F2 worms were screened for Suppression of *wdr-23* (Sow) by bulk sorting worms for loss of bright *Pgst-4*::*GFP* fluorescence in a COPAS BioSorter (Union Biometrica, Holliston, MA). Bulk-sorted F2 worms were then manually screened for viability, and low *Pgst-4*::*GFP* and *Pgst-4*::*tdTomato* fluorescence, and placed into 24-well plates. Sow mutants were also isolated from a selection screen for fast growth and reproduction by four to five rounds of seeding new plates, and allowing them to exhaust the bacteria food source. The largest worms from these plates were isolated and also screened for *Pgst-4*::*GFP* and *Pgst-4*::*tdTomato* fluorescence, and placed into 24 well plates. After ensuring true breeding in F3 progeny, strains were outcrossed to N2. These outcrossed lines were then crossed with Hawaiian mapping strain CB4856, and DNA was isolated from the progeny of recombinant F2s using a ReliaPrep gDNA Tissue Miniprep (Promega Corporation, Madison, WI) supplemented with extra RNAase. Genomic DNA was then labeled and sequenced by the University of Florida Interdisciplinary Center for Biotechnology Research in an Illumina MiSeq (San Diego, CA) according to manufacturer’s recommendations. Raw sequence data were mapped to the N2 reference genome and Hawaiian and N2 SNP variant frequencies and EMS-induced mutations were identified with the CloudMap Workflow on the Galaxy server (Blankenberg *et al.* 2010; Giardine *et al.* 2005; Goecks *et al.* 2010; Doitsidou *et al.* 2010).

### skn-1 transcript analysis

*skn-1* transcripts were amplified with Titanium Taq DNA polymerase (Clontech Laboratories, Mountain View, CA) from cDNA templates. Two exons flanking the *sow*(*zj15*) mutation were Sanger sequenced and analyzed with Geneious 6.0.6 software (Biomatters, New Zealand). Quantitative real-time RT-PCR was used to measure mRNA levels in L4 to young adult stage worms as described previously (Choe *et al.* 2009). Primers for quantitative PCR were: *skn-1b* forward, 5′-CAACAGGTGGATCAACACGG; reverse, 5′-AGGCGTAGTTGGATGTTGGG and *skn-1 a/c* forward, 5′-GGCAAATTTGACCGAGATGCA; reverse, 5′-GAACAAAGTCTCTGGTTGAGCA. Note that the forward primer for *skn-1a/c* spans two exons of the wildtype cDNA, and does not match the sequence of cDNA from mis-spliced mRNA. Primers for quantitative PCR comparison of mis-spliced and normal *skn-1a/c* levels were: *skn-1a/c* normal forward 2, 5′-GTTTATAATCAGGCAAATTTGACCG or *skn-1*(*zj0015*) mis-spliced forward, 5′-AGGCAAATTGCATGTGACCG each paired with *skn-1a/c* reverse, 5′-GAACAAAGTCTCTGGTTGAGCA. Relative starting transcript levels of normal and mis-spliced *skn-1a/c* cDNA were calculated from Ct values and efficiency for each primer pair.

### Generation of transgenic worms

*skn-1* genomic DNA was amplified by PCR with Titanium *Taq* DNA polymerase. PCR products were injected at 5 ng/μl with *Pmyo-2*::*tdTomato* (5 ng/μl) and *Pmyo-3*::*dsRed* (20 ng/μl) as co-markers.

### RNAi

RNAi was performed as described previously (Tang and Choe 2015). dsRNA producing bacteria were grown in Luria-Bertani broth containing selective antibiotic and then transferred to agar nematode growth medium (NGM) plates containing 3 mM isopropyl β-d-1-thiogalactopyranoside (IPTG) or 0.2% β-lactose.

### Growth and reproduction assays

Developmental stage was scored 48 hr after synchronized L1 worms were placed on food. For body length, worms at the same stage (young adult) were photographed, and body length was measured using Image J software (National Institutes of Health). Brood size assays were measured by counting the total number of eggs laid by each worm, and the total number of eggs hatched for 4 d, as described previously (Tang and Choe 2015).

### Fluorescence assays

*Pgst-4*::*GFP* images were taken of adult worms with an Olympus BX60 (Center Valley, PA) microscope fitted with a Zeiss AxioCam MRm camera (Thornwood, NY). Fluorescence intensity was scored manually with a Zeiss Discovery V12 microscope.

### Stress and longevity assays

Stress resistance assays were conducted on worms at L4 to young adult stage, when most of development is complete. Worms were exposed to 5 mM sodium arsenite, or 175 μM juglone. Death was recorded every 2–3 hr for up to 12 hr. For longevity assays, worms were transferred to plates containing 100 mg/ml 5-fluorodeoxyuridine (FUdR) at the L4 to young adult stage. Worms were considered dead if they did not display any movement in response to repeated prodding with a thin platinum wire.

### Statistical analysis

Statistical significance was determined using a Student’s *t*-test when two means were compared, and a one-way ANOVA with a Dunnet or Tukey’s post-hoc test when three or more means were compared, a chi-square test for categorical data, and a log rank test when survival curves were compared. *P* values of < 0.05 were taken to indicate statistical significance, except for comparing more than two survival curves, in which Bonferroni adjustments were made to *P* values to account for repeated comparisons.

### Reagent and data availability

All unique strains and reagents are available on request. Data are available at https://figshare.com/s/006267cd59845048237b.

## Results

### A genetic screen identified a wdr-23 suppressor that mapped to an intron of skn-1

To screen for suppressors of *wdr-23* (Sow), we conducted an EMS screen in *wdr-23*(*tm1817*) worms containing two transcriptional reporters for *gst-4*. Our primary screen was done for suppression of *Pgst-4*::*GFP* fluorescence, which is extremely bright in *wdr-23* worms (Choe *et al.* 2009; Leung *et al.* 2013). This screen is difficult to perform manually because of the inherent difficulty in identifying rare worms with low fluorescence, and therefore a COPAS BioSorter was used. We screened roughly 300,000 F2s derived from 30,000 mutagenized F1s (60,000 genomes). The strain we screened also carried a single copy of *Pgst-4*::*tdTomato* inserted by a MOS transposon method (Frokjaer-Jensen *et al.* 2014); although not bright enough to use in the initial screen, we reasoned that this single copy reporter would allow us to eliminate mutations that only silenced repetitive arrays. After enriching for low GFP fluorescence, we manually screened worms for low tdTomato fluorescence. To isolate additional *sow* mutations, we also screened the same mutagenized lines for suppression of slow growth and reproduction by passing them through at least four generations on agar plates. *wdr-23*(*tm1817*) worms grow and reproduce extremely slowly (Tang and Choe 2015; Choe *et al.* 2009), and therefore suppressor mutations quickly dominated culture plates. Six true-breeding mutant lines were obtained from this selection screen and all but one also had low levels of the *gst-4* reporter fluorescence.

All true-breeding mutant lines with low *gst-4* reporter fluorescence levels were tested for detoxification, and *skn-1* mRNA expression levels using real-time PCR. Two of these mutations [(*zj15*) and (*zj21*)] reduced *skn-1* and target gene (*gst-4*, *gst-10*, and *gst-30*) mRNA levels by more than 50%, and complementation testing suggested that they were not caused by mutations to the same gene.

**Figure 1:**
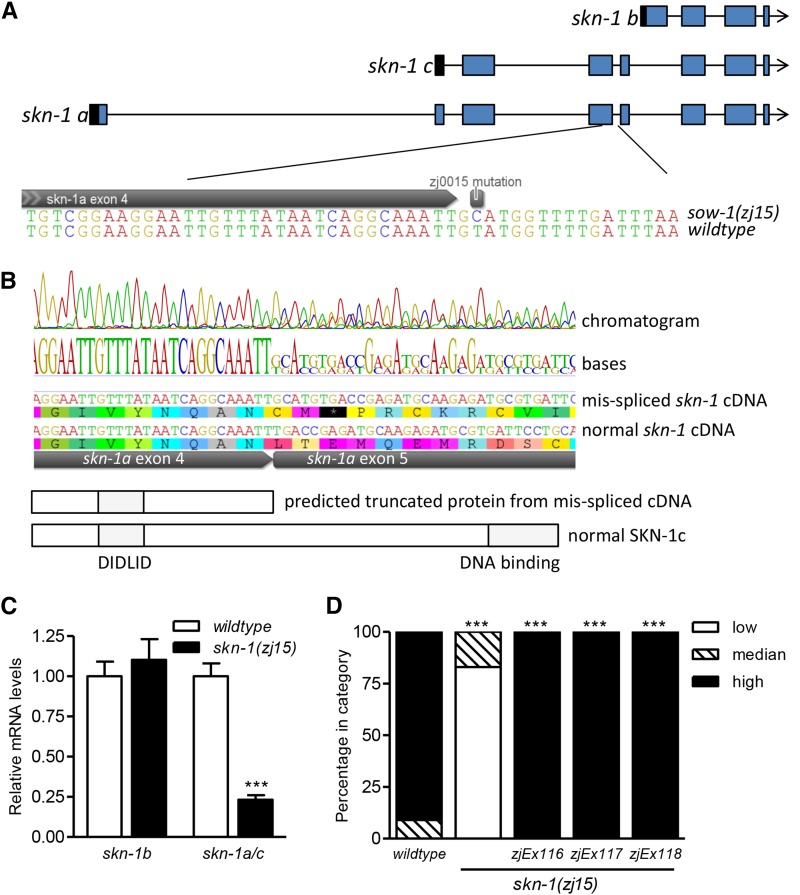
*sow-1*(*zj15*) maps to an intron of *skn-1*. (A) Schematic diagram of three *skn-1* splice variant gene models; the mutated base is shown relative to exon 4 of *skn-1a*. (B) Direct RT-PCR product sequence results (top) and predicted proteins (bottom). *sow-1*(*zj15*) is an AT–GC mutation in an intron that is specific to *skn-1a* and *c*. Sequencing of cDNA reveals that a fraction of *skn-1a/c* transcripts have a splicing error that introduces an extra 5 bp (GCATG) from the beginning of this intron. The consequence of this spicing error is the addition of two amino acids and a stop codon that removes the entire c-terminus and DNA binding domain. (C) mRNA levels of *skn-1b* and *skn-1a/c* transcripts (mean ± SE, *n* = 5 replicates of worms, ***P < 0.001 relative to wildtype). (D) Rescuing effects of *skn-1* gDNA on *Pgst-4*::*GFP* expression during exposure to 2.8 mM acrylamide [*n* = 29–78 total worms, ***P < 0.001 relative to *sow-1*(*zj15*)]; given full rescue, we conclude that *sow-1* is *skn-1*, and refer to the allele as *skn-1*(*zj15*) from this point forward.

Both alleles were crossed with the Hawaiian mapping strain CB4856, and the genomic DNA of 59 (*zj15*) or 43 (*zj21*) recombinants was sequenced to an average coverage of 51X (*zj15*) or 41X (*zj21*) and mapped. *sow-1*(*zj21*) mapped to two regions, one corresponding to an intron mutation in *skn-1*, and the other to a broad (6–7 Mb) region of chromosome III, suggesting that at least two mutations are contributing to the phenotype; this allele was not characterized further. Alternatively, *sow-1*(*zj15*) mapped to a 2-Mb region of chromosome IV that includes an AT to GC mutation in the fourth intron of the longest *skn-1* variant (Figure 1A).

Direct Sanger sequencing of an RT-PCR product corresponding to this region indicates that two pools of *skn-1a/c* transcript are produced in *sow-1*(*zj15*) worms, some spliced normally, and some with an extra 5 bp from the mutated intron that causes a frame shift predicted to result in a truncated protein (251/533 amino acids for SKN-1c) that is missing all of the DNA binding domain (Figure 1B). We next used quantitative PCR to determine the mRNA levels of *skn-1* variants. The mRNA level of *skn-1b* was not changed by *sow-1*(*zj15*) (*P* = 0.5358), but *skn-1a/c* mRNA was reduced 76% compared to wild type (*P *< 0.0001) (Figure 1C). We also designed primers specific for each version of *skn-1a/c* cDNA (mis-spliced and wildtype). In three cDNA samples from *sow-1*(*zj15*), we found that the wildtype and mis-spliced variants are present at an average ratio of 1.00 ± 0.12 to 0.65 ± 0.04, respectively.

We next tested if transgenic expression of wildtype *skn-1* genomic DNA could rescue *Pgst-4*::*GFP* induction in the *sow-1*(*zj15*) mutant. We exposed animals to 2.8 mM acrylamide to induce *Pgst-4*::*GFP*. As expected, *sow-1*(*zj15*) abolished the induction of *Pgst-4*::*GFP* (Figure 1D). Transgenic expression of wildtype *skn-1* genomic DNA completely rescued *Pgst-4*::*GFP* expression to wildtype levels in three independent lines (Figure 1D). We conclude that the phenotype causing mutation is within *skn-1*, and refer to it as *skn-1*(*zj15*) from this point forward.

### Some skn-1(zj15) worms escape embryonic lethality

**Figure 2:**
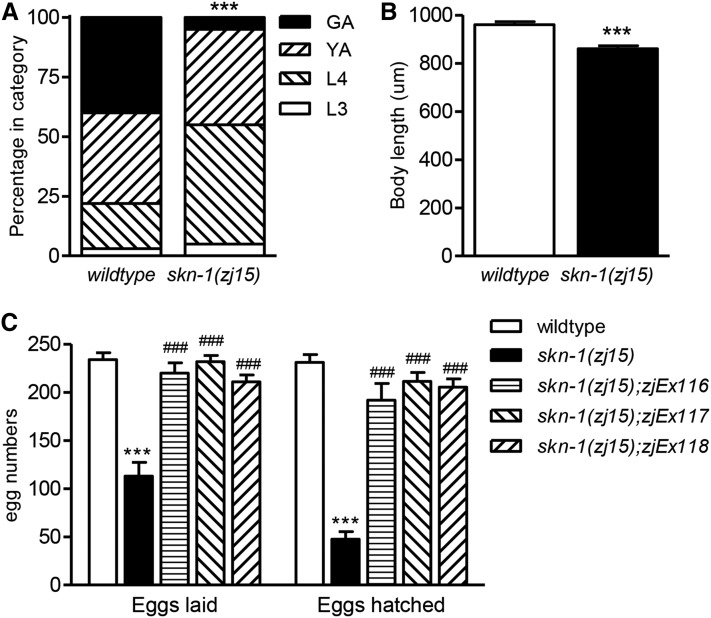
*skn-1*(*zj15*) worms are viable. (A) Developmental stages of wildtype and *skn-1*(*zj15*) worms grown from synchronized L1 for 2 d (*n* = 193–303 total worms, ***P < 0.001 relative to wildtype). (B) Body length of worms at the young adult stage (*n* = 30–37 total worms, ***P < 0.001 relative to wildtype). (C) Numbers of total eggs and hatched eggs produced from individual hermaphrodites [mean ± SE, *n* = 6–12 total worms, ***P < 0.001 relative to wildtype, ^###^*P* < 0.001 relative to *skn-1*(*zj15*)].

To our knowledge, all current loss-of-function *skn-1* alleles are 100% penetrant maternal effect embryonic lethal. Alternatively, homozygote *skn-1*(*zj15*) worms appear superficially wildtype and reproduce and grow, indicating that at least some are viable. Larval developmental rate (Figure 2A) and body length (Figure 2B) were slightly decreased compared to wildtype N2 worms when *skn-1*(*zj15*) worms were measured. As shown in Figure 2C, *skn-1*(*zj15*) worms laid about half as many eggs as wildtype, and 42% of the eggs hatched. Therefore, each *skn-1*(*zj15*) worm produced an average of about 47 viable offspring. We next tested if transgenic expression of wildtype *skn-1* genomic DNA could rescue the egg production and partial embryonic lethality phenotypes of *skn-1*(*zj15*); total eggs laid and number hatched were both rescued to nearly wildtype levels (Figure 2C), suggesting that the *skn-1* mutation is responsible for both phenotypes.

### skn-1(zj15) is functionally comparable to skn-1 RNAi

**Figure 3:**
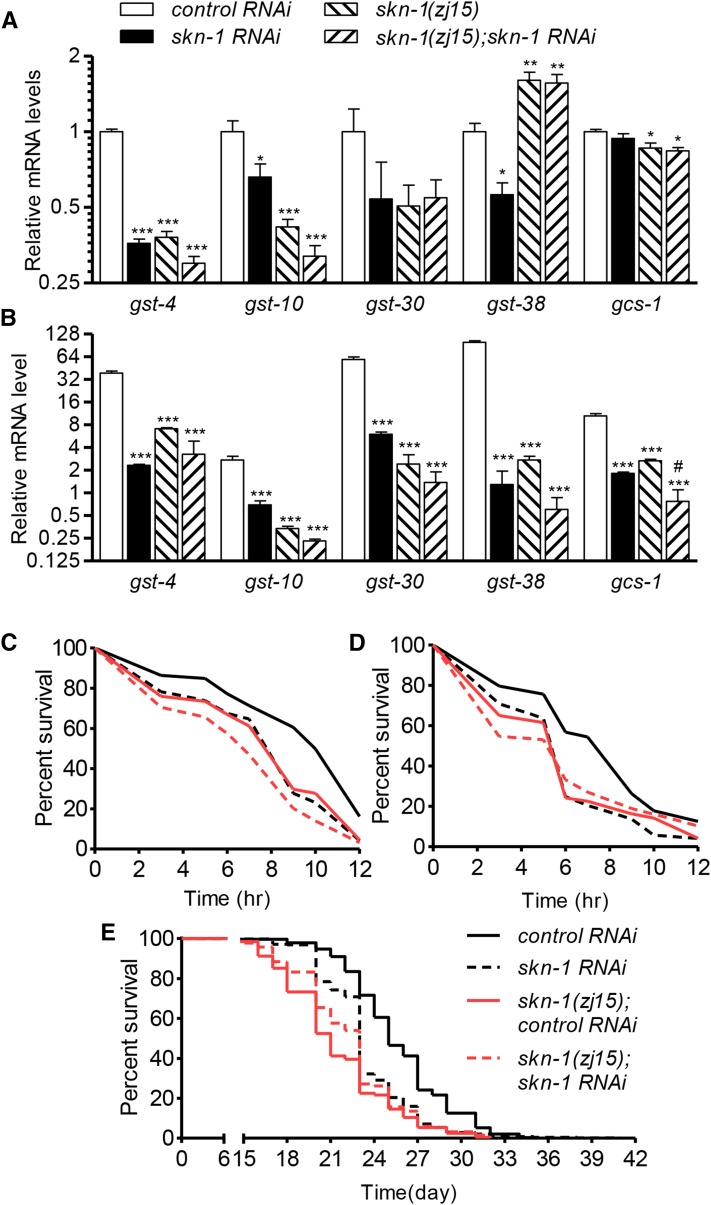
*skn-1*(*zj15*) is functionally comparable to *skn-1* RNAi. (A) Relative mRNA levels under basal conditions (mean ± SE, *n* = 5 replicates of worms, ***P < 0.001, **P < 0.01, *P < 0.05 relative to control RNAi). (B) Relative mRNA levels after exposure to 5 mM As for 1 hr [mean ± SE, *n* = 4–5 replicates of worms, ***P < 0.001 relative to control RNAi, and ^#^*P* < 0.05 relative to *skn-1*(*zj15*)]. (C) Survival of 5 mM arsenite [*n* = 242–295 total worms from three trials, *P* < 0.0001 for control RNAi relative to *skn-1* RNAi or *skn-1*(*zj15*), *P* = 0.7716 for *skn-1* RNAi *vs.* *skn-1*(*zj15*), *P* = 0.0005 for *skn-1*(*zj15*) *vs.* *skn-1*(*zj15*); *skn-1 RNAi*, *P* < 0.0125 was taken to indicate statistical significance]. (D) Survival of 175 µM juglone [*n* = 252–322 total worms from three trials, *P* < 0.0001 for control RNAi relative to *skn-1* RNAi or *skn-1*(*zj15*), *P* = 0.4792 for *skn-1* RNAi *vs.* *skn-1*(*zj15*), *P* = 0.2967 for *skn-1*(*zj15*) *vs.* *skn-1*(*zj15*); *skn-1 RNAi*, *P* < 0.0125 was taken to indicate statistical significance]. (E) Lifespan analysis [*n* = 290–487 total worms from three trials, *P* < 0.0001 for control RNAi relative to *skn-1* RNAi or *skn-1*(*zj15*), *P* < 0.0001 for *skn-1* RNAi *vs.* *skn-1*(*zj15*), *P* = 0.0001 for *skn-1*(*zj15*) *vs.* *skn-1*(*zj15*); *skn-1* RNAi, *P* < 0.0125 was taken to indicate statistical significance]. Note that the survival trials in Figure 3, C, D, and E were run with those in Figure 5, C, D, and E, respectively, and that the control curves are the same.

To determine if *skn-1*(*zj15*) mutants are functionally comparable to *skn-1* RNAi with respect to stress response, we measured the mRNA levels of core SKN-1 target genes (*gst-4*, *gst-10*, *gst-30*, *gst-38*, and *gcs-1*) under basal and stress-induced conditions. The basal mRNA levels of *gst-4*, *gst-10*, and *gcs-1* were lower in *skn-1*(*zj15*) mutants to an extent similar to, or more than, with *skn-1* RNAi (Figure 3A). *skn-1* RNAi in *skn-1*(*zj15*) worms did not further decrease the expression of any of the target genes. Exposure to 5 mM sodium arsenite for 1 hr induced expression of all the target genes in wild-type worms (Figure 3B), and *skn-1* RNAi or *skn-1*(*zj15*) largely suppressed their induction; *skn-1* RNAi in *skn-1*(*zj15*) worms did not further decrease the expression of any target genes except for *gcs-1* (*P* *<* 0.05).

We also characterized the effects of *skn-1*(*zj15*) on oxidative stress resistance and longevity. As expected, *skn-1* RNAi decreased survival on both 5 mM sodium arsenite and 175 μM juglone; the effect of *skn-1*(*zj15*) was similar [*skn-1* RNAi *vs.* *skn-1*(*zj15*): *P* = 0.7716 for sodium arsenite, *P* = 0.4792 for juglone) (Figure 3, C and D). *skn-1* RNAi in *skn-1*(*zj15*) worms slightly decreased survival to sodium arsenite (*P* = 0.0005) but not to juglone (*P* = 0.2967) (Figure 3, C and D). Both *skn-1* RNAi and *skn-1*(*zj15*) decreased lifespan (*P* < 0.0001 for both), but *skn-1*(*zj15*) had a larger effect (median lifespans of 23 and 21 d, respectively, *P* < 0.0001) (Figure 3E); *skn-1* RNAi in *skn-1*(*zj15*) worms did not further reduce longevity (Figure 3E).

### skn-1(zj15) suppresses gst-4 transcription in tissues that are resistant to RNAi

**Figure 4:**
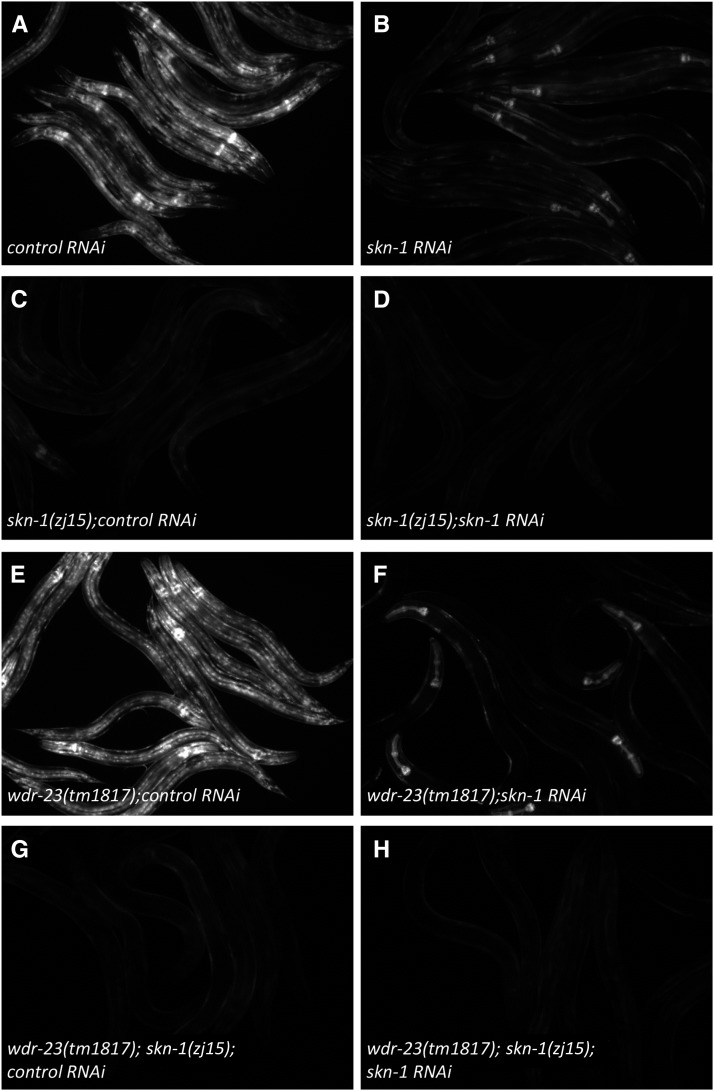
*skn-1*(*zj15*) suppressed *gst-4* transcription in tissues that are resistant to RNAi. All panels are micrographs of *Pgst-4*::*GFP* fluorescence in at least seven worms. (A–D) Worms of the indicated genotypes were constitutively grown on plates containing 2.8 mM acrylamide for 48 hr. (E–H) Worms of the indicated genotypes were imaged.

RNAi in *C. elegans* suffers from being refractory in some tissues (Kamath *et al.* 2001; Asikainen *et al.* 2005). We compared inhibition of *Pgst-4*::*GFP* fluorescence induced by acrylamide between *skn-1* RNAi and *skn-1*(*zj15*). Acrylamide (2.8 mM) strongly induced *Pgst-4*::*GFP* fluorescence in many tissues (Figure 4A), and *skn-1* RNAi largely suppressed this induction in most tissues except for the pharynx and body wall muscle (Figure 4B). In contrast, *skn-1*(*zj15*) suppressed GFP in all tissues (Figure 4C). *skn-1* RNAi further inhibited *Pgst-4*::*GFP* fluorescence slightly in *skn-1*(*zj15*) worms (observed manually, but difficult to see in Figure 4D). We also compared the effects of *skn-1* RNAi and *skn-1*(*zj15*) on the inhibition of *Pgst-4*::*GFP* in *wdr-23*(*tm1817*) mutants. Consistent with what we observed with acrylamide, *skn-1*(RNAi) was refractory in the pharynx and body wall muscle, and *skn-1*(*zj15*) suppressed *Pgst-4*::*GFP* fluorescence in all tissues (Figure 4, E–H).

### skn-1(zj15) is epistatic to pathways upstream of SKN-1

**Figure 5:**
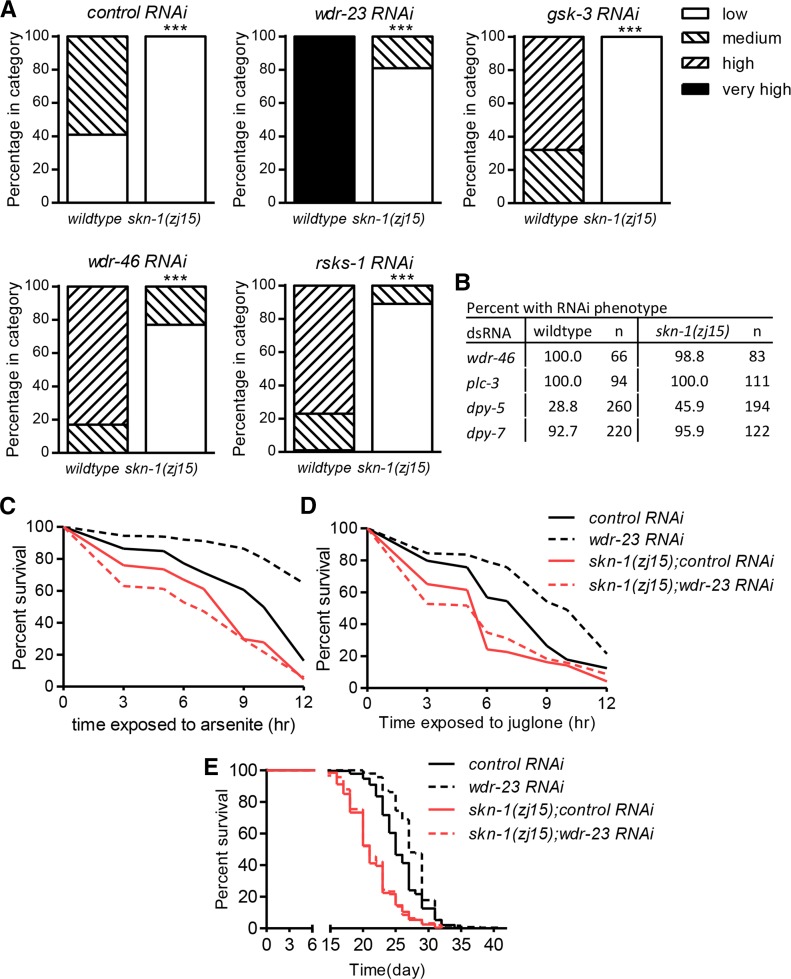
*skn-1*(*zj15*) is a tool for epistasis. (A) *skn-1*(*zj15*) suppressed *Pgst-4*::*GFP* induction by loss of known inhibitory signals upstream of SKN-1. ***P < 0.001 relative to wildtype. (B) RNAi phenotype scoring for sterility (*wdr-46* and *plc-3*) and dumpy (*dpy-5* and *dpy-7*) in *skn-1*(*zj15*) worms. (C) Survival of 5 mM arsenite [*n* = 214–251 total worms from three trials, *P* < 0.001 for control RNAi relative to *wdr-23* RNAi or *skn-1*(*zj15*), *P* < 0.001 for *wdr-23* RNAi *vs.* *skn-1*(*zj15*); *wdr-23* RNAi, *P* = 0.0654 for *skn-1*(*zj15*) *vs.* *skn-1*(*zj15*); *wdr-23* RNAi, *P* < 0.0125 was taken to indicate statistical significance]. (D) Survival of 175 µM juglone [*n* = 252–353 total worms from three trials, *P* < 0.001 for control RNAi relative to *wdr-23* RNAi or *skn-1*(*zj15*), *P* < 0.001 for *wdr-23* RNAi *vs.* *skn-1*(*zj15*); *wdr-23* RNAi, *P* = 0.490 for *skn-1*(*zj15*) *vs.* *skn-1*(*zj15*); *wdr-23* RNAi, *P* < 0.0125 was taken to indicate statistical significance]. (E) Lifespan analysis [*n* = 278–462 total worms from three trials, *P* < 0.001 for control RNAi relative to *wdr-23* RNAi or *skn-1*(*zj15*), *P* < 0.001 for *wdr-23* RNAi *vs.* *skn-1*(*zj15*); *wdr-23* RNAi, *P* = 0.7337 for *skn-1*(*zj15*) *vs.* *skn-1*(*zj15*); *wdr-23* RNAi, *P* < 0.0125 was taken to indicate statistical significance]. Note that the survival trials in Figure 3, C, D, and E were run with those in Figure 5, C, D, and E, respectively, and that the control curves are the same.

SKN-1 is regulated by diverse signaling pathways (Tullet *et al.* 2008; An *et al.* 2005; Li *et al.* 2011; Robida-Stubbs *et al.* 2012; Choe *et al.* 2009). As proof-of-principle as a tool for genetic interaction studies, we tested if *skn-1*(*zj15*) is epistatic to known signaling components upstream of SKN-1 including a nucleolar protein (WDR-46) (Choe *et al.* 2009), glycogen synthase kinase-3 (GSK-3) (An *et al.* 2005), and a putative ribosomal protein S6 kinase (RSKS-1) (Robida-Stubbs *et al.* 2012). We used RNAi to knock down the expression of these signaling genes, and scored *Pgst-4*::*GFP* fluorescence. RNAi of *wdr-23*, *wdr-46*, *gsk-3*, and *rsks-1* all induced *Pgst-4*::*GFP*, and *skn-1*(*zj15*) strongly suppressed the induction of *Pgst-4*::*GFP* fluorescence under all these conditions (Figure 5A). Scoring of RNAi sterility (*wdr-46* RNAi and *plc-3* RNAi) and body morphology (*dpy-5* RNAi and *dpy-7* RNAi) phenotypes not associated with *skn-1* did not detect any evidence of an RNAi defective phenotype in *skn-1*(*zj15*) worms (Figure 5B).

WDR-23 is a principal and direct suppressor of SKN-1 (Choe *et al.* 2009). Loss of function of *wdr-23* increases stress resistance and longevity in a *skn-1*-dependent manner (Tang and Choe 2015). Here, we tested if *skn-1*(*zj15*) could suppress these *wdr-23* phenotypes. As expected, *wdr-23* RNAi strongly increased survival of 5 mM sodium arsenite and 175 μM juglone (Figure 5, C and D). The increased survival phenotypes were abolished in *skn-1*(*zj15*) mutants. Similarly, *wdr-23* RNAi failed to increase lifespan in *skn-1*(*zj15*) mutants (Figure 5E). Note that the survival trials in Figure 3, C, D, and E were run with those in Figure 5, C, B, and E, respectively, and that the control curves are the same.

## Discussion

In summary, we isolated a new *skn-1* hypomorphic allele *skn-1*(*zj15*) that can be maintained as a homozygote without a genetic balancer. The effects of *skn-1*(*zj15*) on expression of SKN-1 target genes, stress resistance, and longevity were all similar to, or stronger than, *skn-1* RNAi except that the effects of the allele were not limited by tissue type like RNAi (Figure 3 and Figure 4). Importantly, *skn-1* RNAi did slightly enhance *gst-4* reporter and survival phenotypes of *skn-1*(*zj15*) worms, consistent with residual functional SKN-1 being expressed. This is also reflected in the production of close to 50 viable offspring per homozygous hermaphrodite (Figure 2C), compared to none from worms homozygous for currently available nonsense *skn-1* alleles (WormBase 2015).

SKN-1 regulation and function are under intense investigation and epistasis is a useful tool for placing genes into ordered genetic pathways (Tullet *et al.* 2008; An *et al.* 2005; Li *et al.* 2011; Robida-Stubbs *et al.* 2012; Blackwell *et al.* 2015). The ability of *skn-1*(*zj15*) to suppress known upstream regulators (Figure 5) demonstrates its utility as a tool for epistasis analysis without the technical problems inherent with nonsense mutation alleles that require balancers. We conclude that *skn-1*(*zj 0015*) is a strong hypomorphic allele with stress and longevity phenotypes as strong as, or stronger than, *skn-1* RNAi. We propose that this allele could be used in cases where food sources are varied, constant dsRNA feeding is not appropriate, epistasis with an RNAi phenotype is needed, or when a large population is required. Potential users should, however, keep in mind that *skn-1*(*zj15*) is not a null allele when interpreting genetic interaction results as they would when using *skn-1* RNAi.

The 76% reduction in wildtype *skn-1a/c* mRNA (Figure 1) alone could cause the stress response and embryonic lethality phenotypes in *skn-1*(*zj15*) homozygotes. During outcrossing and mapping, *skn-1*(*zj15*) heterozygotes had strong *Pgst-4*::*GFP* induction on plates with acrylamide, consistent with the mutation being recessive for the stress response phenotype. However, we cannot rule out the possibility of truncated SKN-1 contributing in a dominant manner in *skn-1*(*zj15*) homozygotes, because the ratio of normal to mis-spliced SKN-1 protein is likely to be different than in heterozygotes.

Surprisingly, homozygous *skn-1*(*zj15*) worms have a total egg production defect that was not observed with a nonsense *skn-1* allele or RNAi (Tang and Choe 2015). Rescue of this defect with wildtype *skn-1* genomic DNA is consistent with this phenotype being caused by *skn-1*(*zj15*) and not a background mutation. It is unclear how *skn-1*(*zj15*) influences egg production, and care should be taken when interpreting reproductive phenotypes with this allele.
